# Lyn is involved in CD24-induced ERK1/2 activation in colorectal cancer

**DOI:** 10.1186/1476-4598-11-43

**Published:** 2012-06-26

**Authors:** Ning Su, Liang Peng, Bingqing Xia, Yingying Zhao, Angao Xu, Jing Wang, Xinying Wang, Bo Jiang

**Affiliations:** 1 Department of Gastroenterology, Nanfang Hospital, Southern Medical University, Guangzhou, 510515, China; 2 Guangdong Provincial key laboratory of Gastroenterology, Guangzhou, 510515, China; 3 Huizhou Medical Institute, Huizhou, Guangdong, 516001, China

**Keywords:** CD24, Lyn, ERK1/2, Colorectal cancer

## Abstract

**Background and aim:**

CD24 expression is associated with human colorectal cancer (CRC). Our previous data indicated that CD24 promoted the proliferation and invasion of colorectal cancer cells through the activation of ERK1/2. Since Src family kinases are frequently deregulated in CRC and closely related to the MAPK signaling pathway, we investigated the impact of Lyn, an important member of SFKs, on CD24-induced ERK1/2 activation in CRC.

**Methods and Results:**

The interaction of CD24 and Lyn was identified by co-immunoprecipitation (Co-IP) and ectopic expression of CD24-induced Lyn activation. Inhibition of Lyn activation by phosphatase PP2 in SW480^CD24^cells abrogated CD24-induced invasion. The results of the Co-IP and immunofluorescence assay revealed that overexpression of CD24 enhanced the interaction of Lyn and ERK1/2 and induced the nuclear translocation of Lyn. However, inhibition of Lyn activity attenuated CD24-induced ERK1/2 activation, and depletion of CD24 disrupted Lyn-ERK1/2 interaction. Immunohistochemistry analysis for 202 cases of CRC showed that the expression of both CD24 and Lyn was positively correlated with tumor grade, stage, lymph node and distant metastasis. Patients with lower expression of CD24 or Lyn had a higher survival rate. The Cox multivariate analysis showed that CD24 expression, but not Lyn expression, was an independent prognostic factor of CRC.

**Conclusions:**

Our results suggest that Lyn is involved in CD24-induced ERK1/2 activation in CRC. The expression of CD24 is associated with activation of Lyn and ERK1/2, which might be a novel mechanism related to CD24-mediated regulation of CRC development.

## Background

CD24, a small glycosyl-phosphoinositol-anchored membrane protein ranging from 30–70 kDa, consists of a small protein core comprising 27 amino acids, with several potential O- or N-linked glycosylation sites [1]. CD24 has been identified to be up-regulated in various solid tumors, such as breast cancer [2], prostate cancer [3] and cholangiocarcinoma [4], and its overexpression is usually associated with poor clinical outcomes in some tumor types [2-4]. Chou *et al.*[5] reported strong cytoplasmic CD24 expression in diffuse- or mixed-type gastric adenocarcinomas. In addition, Sagiv *et al.*[6] identified that increased expression of CD24 was an early event in the carcinogenesis of colorectal cancer (CRC). Our previous study also revealed that CD24 expression occurred in 92.5 % of human CRC tissue and increased with tumor progression [7]. Furthermore, we showed that CD24 played an important role in the carcinogenesis of CRC.

CD24 is a short mucin-like peptide present at the outer cell surface and lacks a cytoplasmic domain to transduce intracellular signals. However, CD24 has been identified as a novel regulator of proliferation [7], apoptosis [8] and invasion [9] in human cancer. Several ligands of CD24, including P-selectin [10] and Siglec-10 [11], have been identified and are found to be crucial for tumor development. In our previous study, we found that the activation of extracellular signal-regulated kinases 1 and 2 (ERK1/2) and p38 MAPK were dependent of CD24 and required for the proliferation [7] and invasion of CRC cells *in vitro* and *in vivo* (unpublished data). Although CD24 is an important player in CRC, the mechanisms of its function in CRC remain unclear. Exploring the mechanisms underlying CD24-mediated activation of MAP kinases would be beneficial in for better understanding of the role of CD24 in CRC development.

To this end, the connection between CD24 and MAP kinases in literature has been studied. Zarn *et al.* found that CD24 localized in glycolipid-enriched membrane (GEM) domains, which are the specialized areas in the plasma membrane signaling platforms, and associated with Lyn in an erythroleukemia cell line [12]. Moreover, Petra *et al.* showed that CD24 interacted with c-Src and promoted its activity within lipid rafts in breast cancer cells [13]. Furthermore, many studies have suggested that Src family kinases (SFKs) are located upstream of MAPKs cascades in several receptor signaling systems [14]. SFKs are a family of non receptor-type tyrosine kinases and include at least nine highly homologous proteins in mammals [15,16]. Lyn is an important member of the SFKs and widely expressed in B-lymphocytes and myeloid cells. Lyn establishes thresholds by acting as both a positive and negative modulator of a variety of signaling responses [17]. Furthermore, aberrant activation of Lyn has been implicated in variety of human tumors, including breast cancer [18,19], prostate cancer, glioblastoma and CRC [20,21]. Therefore, we hypothesize that SFKs are involved in the CD24-induced ERK1/2 activation. In the present study, we examined the correlation between CD24 and Lyn in CRC. Our results revealed that CD24 interacted with Lyn and induced the activation and nuclear translocation of Lyn. In contrast, the inactivation of Lyn abrogated CD24-induced cell invasion and ERK1/2 activation in CRC cells. Analysis of CRC tissues with immunohistochemistry staining showed that the expression of CD24 and Lyn was positively correlated and associated with tumor stage and lymph node and distant metastasis. Our study suggests that the expression of CD24 is associated with the activation of Lyn and ERK1/2, which may be a novel mechanism related to CD24-mediated regulation of CRC development.

## Results

### Lyn interacted with CD24 and was activated by CD24 in CRC cells

**Figure 1:**
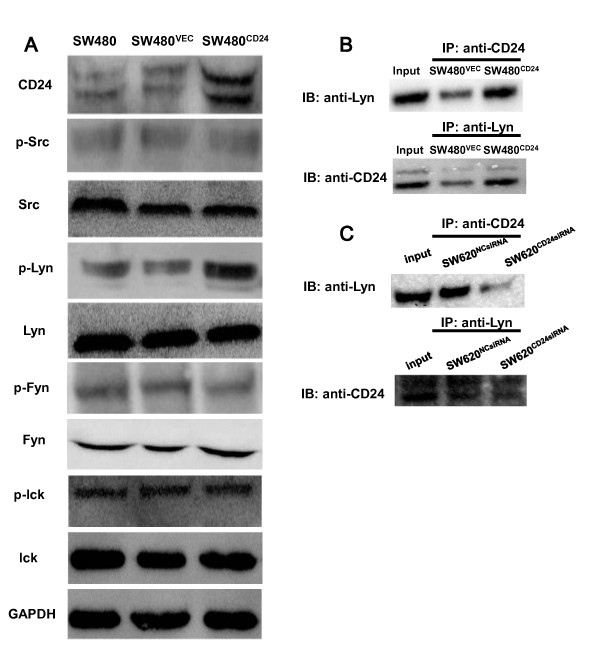
**Interaction between CD24 and Lyn. A.** Immunoblot analysis for phosphorylation levels of Src, Lyn, Fyn and lck in SW480^CD24^ and vehicle control cells. GAPDH served as an internal control. All of these pictures were representatives of three independent experiments. **B.** The interaction between Lyn and CD24 was examined in SW480^CD24^ (lane 2) and SW480^VEC^ (lane 3) cells. Immunoprecipitation was performed with an anti-CD24 antibody, and normal rabbit immunoglobulin G (Ig G) served as a control. Western blotting was performed with an anti-Lyn antibody. Input (lane 1) is 1 % of the extract from untreated cells. **C.** CD24 was depleted with CD24 specific siRNA in SW620 cells. Cell lysates were immunoprecipitated with an anti-CD24 antibody and Western blotting was performed with anti-Lyn antibody.

To investigate the association of CD24 and SFKs, we examined the activation of SFKs, including Src, Lyn, Fyn and lck in SW480^CD24^ cells and SW480 vector and parental cells. The results showed that ectopic expression of CD24 increased the phosphorylation level of Lyn, but not Src, Fyn or lck (Figure 1A), suggesting that CD24 specifically induced the activation of Lyn, which is a signaling molecule anchoring to plasma membrane. The densitometry results of Figure 1A are shown in Additional file 1: Figure S1. To determine whether there was an interaction between CD24 and Lyn, we performed a CO-IP assay. The results revealed that endogenous or ectopic Lyn were co-precipitated with CD24 in both SW480^VEC^ and SW480^CD24^ cells when using an anti-Lyn antibody. This interaction was also confirmed in the reciprocal immunoprecipitation with anti-CD24 antibody (Figure 1B), indicating that endogenous CD24 was capable of binding to Lyn, but binding was weaker than that of the ectopic CD24. Furthermore, the increasing amount of immuneprecipitates in SW480^CD24^ cells suggested that the overexpression of CD24 might promote the CD24-Lyn interaction in a direct or indirect manner. Consistent with this observation, the depletion of CD24, using a specific siRNA in SW620 cells, significantly reduced immunoprecipitates in the CO-IP assay (Figure 1C).

### Ectopically expressed CD24 promoted cell invasion and induced Lyn activation and translocation into the nucleus

**Figure 2:**
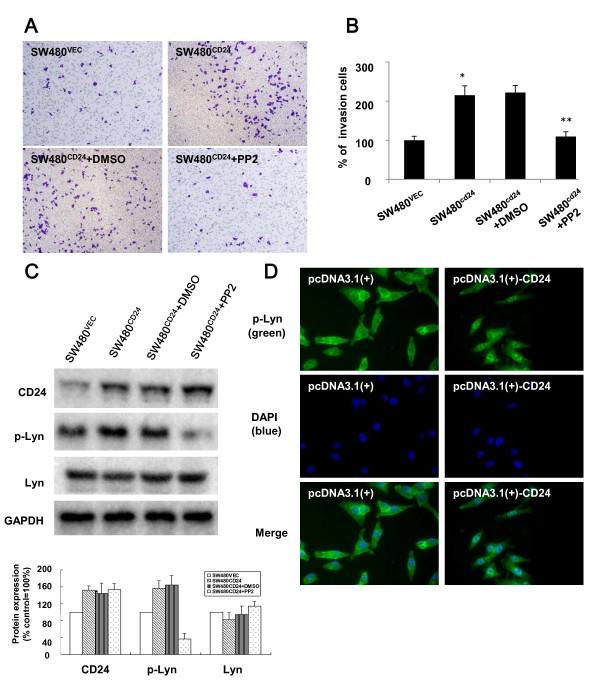
**Inhibition of Lyn activation attenuated CD24-induced CRC cell invasion. A.** Representative images from the cell invasion assay. SW480^CD24^ cells or vehicle control cells were seeded into transwell chambers coated with Matrigel and 10 μM PP2 was added 24 h later and kept for 30 min. The cell invasion assay was performed at 24 h after PP2 treatment. CD24 increased the capacity of SW480 cells to migrate through the filters compared with the controls cells (upper). Furthermore, the migration capacity of the SW480^CD24^ cells that were treated by PP2 (10 μM) was inhibited (bottom). **B.** Quantification of cell invasion. Invasive activity was determined as the percent invasion of the control SW480 cells through the Matrigel membrane. Values shown are means *±* standard errors (SE) for three independent experiments. * P < 0.05 between SW480^VEC^ and SW480^CD24^ cells; ** P < 0.05 between (SW480^CD24^ + DMSO) and (SW480^CD24^ + PP2) cells. **C.** Western blotting analysis showed the phosphorylation of Lyn was induced by the over-expression of CD24 (lane 2) and the activation of Lyn was inhibited by PP2 (lane 4). GAPDH served as a loading control. Bar graph depicted the quantitative analysis for protein expression and the expression of each protein was normalized to GAPDH. **D.** Immunofluorescent staining of cells transfected with a pcDNA3.1(+)-CD24 plasmid. SW480 cells were transfected with a CD24 expression plasmid or vehicle control. After 24 h, cells were fixed, permeabilized and stained with 4,6-diamidino-2-phenylindole (DAPI; blue) and Lyn (green). In the cells with overexpressed CD24, Lyn was activated. phospho-Lyn was more intense in the nucleus.

We next examined the effect of ectopic expression of CD24 by transfection with a pcDNA3.1(+)-CD24 expression plasmid [7] on the invasion capability of SW480 cells using a cell invasion kit. Invasion capability was quantified as the percentage of invasive cells compared to the total number cells. Representative images of invasive cells of each group are shown in Figure 2A (upper, original magnification, 200×). We observed a 2.15-fold increase in cellular invasion in cells transfected with the pcDNA3.1(+)-CD24 plasmid compared with the control cells (Figure 2B) (p < 0.05). The expression levels of CD24 and the phosphorylation of Lyn were assessed by Western blot analysis. There was an increase in Lyn phosphorylation (Y396) in SW480^CD24^ cells as compared to SW480^VEC^ cells (p < 0.05) (Figure 2C). To investigate the role of Lyn in CD24-mediated cell invasiveness, cells were treated with 10 μM PP2 and tested using a cell invasion assay. The percentage of cells that migrated through the filters from different groups was shown in Figure 2A and B. Similar results were obtained in SW620 cells (data not shown).

Since previous studies showed that distinct localization of Lyn was critical for its function, the cellular localization of Lyn was examined by analysis of dual immunofluorescence staining in this study. As shown in Figure 2D, endogenous phospho-Lyn (green) in control cells was weak and expressed throughout the entire cells (left panels), whereas phospho-Lyn in CD24 expressing cells was more prominent and more intense in the nucleus (right panels). These findings suggested that CD24 could induce Lyn activation and translocation into the nucleus.

### Immunostaining of Lyn strongly correlated with CD24 expression in human CRC tissues and cancer progression

**Figure 3:**
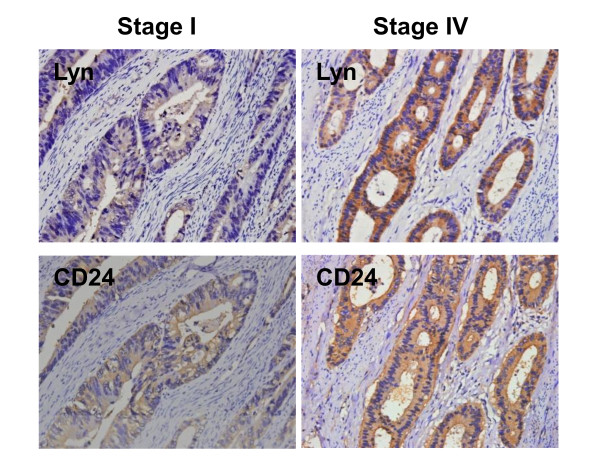
**The representative pictures of immunohistochemical staining for Lyn and CD24 in serial sections of CRC tissues with different stages (left: stage I; right: stage IV).** (original magnification = 400×). Lyn (upper) and CD24 (bottom) showed weak expression in primary cancer tissues (stage I) and strong expression in metastatic cancer tissues (stage IV).

To confirm the correlation between CD24 and Lyn *in vivo*, immunohistochemical staining was performed in serial sections of human CRC tissues. Lyn and CD24 staining were present mainly in the membrane and/or cytoplasm (Figure 3) and were positively correlated (r = 0.443, p < 0.05) (Table 1).

**Table 1:** The correlation of CD24 and Lyn expression in 202 CRC patients

**CD24**	**Lyn**				**r**	***P***
	**-**	**+**	**++**	**+++**		
**-**	4	2	0	0	0.443	0.000
**+**	6	72	12	8		
**++**	2	18	54	6		
**+++**	2	6	6	4		

Immunohistochemical staining of the CRC tissue showed that CD24 and Lyn had different degrees of staining. For CD24 staining, 51 % of the CRC tissues showed positive and 49 % showed moderate or strongly positive staining, while its expression in adjacent normal mucosa was either absent or barely detectable. For Lyn staining, 56 % of the CRC tissure showed positive and 44 % showed moderately or strongly positive staining, while its expression in adjacent normal mucosa was either absent or barely detectable.

There was a significantly positive correlation between CD24 expression and tumor grade, tumor stage, invasion depth and lymph node and distant metastasis (p < 0.05), while no correlation was observed between CD24 expression and age, sex, localization and tumor size (Figure 3 and Table 2). Lyn expression correlations were similar to CD24 expression. Our results showed that CD24 and Lyn expression increased with tumor progression.

**Table 2:** Correlation of CD24 and Lyn expression and clinic-pathological parameters in 202 CRC patients

		**CD24**							**Lyn**						
**Item**	**Case**	**-**	**+**	**++**	**+++**	**Mean ranks**	z/ $\chi_{}^{2}$/r	***P***	**-**	**+**	**++**	**+++**	**Mean ranks**	z/ $\chi_{}^{2}$/r	***P***
*Gender															
Male	110	2	58	38	12	100.35	−0.336	0.737	9	54	38	9	98.92	−0.749	0.454
Female	92	4	40	42	6	102.87			5	44	34	9	104.59		
*Age(years)															
≤60	112	4	57	45	6	96.79	−1.407	0.159	8	56	40	8	99.14	−0.698	0.485
>60	90	2	41	35	12	107.36			6	42	32	10	104.43		
^#^Localization															
Right colon	32	1	15	14	2	101.44	0.02	0.990	2	19	8	3	93.44	1.217	0.544
Left colon	68	1	34	29	4	100.81			4	30	27	6	106.05		
Rectum	102	4	49	37	12	101.98			8	49	37	9	101.04		
*Tumor size															
≤5 cm	155	4	78	58	15	100.82	−0.333	0.739	9	80	54	12	99.93	−0.759	0.448
>5 cm	47	2	20	22	3	103.76			5	18	18	6	106.69		
^&^Grade															
G1	99	3	58	34	4		0.255	0.000	6	58	30	5		0.233	0.000
G2	64	2	30	26	6				6	31	23	4			
G3	39	1	10	20	8				2	9	19	9			
^&^Tumor stage															
I	30	2	22	6	0		0.577	0.000	3	20	5	4		0.399	0.000
II	85	2	62	20	1				8	59	17	3			
III	50	1	12	32	5				2	12	31	3			
IV	37	1	2	22	12				1	7	19	8			
^&^Invasion depth															
T1	38	2	24	10	2		0.293	0.000	5	22	9	2		0.297	0.000
T2	98	1	55	37	5				4	58	30	6			
T3	44	2	17	22	3				4	16	20	4			
T4	22	1	2	11	8				1	2	13	6			
*Lymph node metastasis															
Negative	130	5	84	38	3	82.70	−6.770	0.000	11	81	31	7	86.03	−5.516	0.000
Positive	72	1	14	42	15	135.44			3	17	41	11	129.43		
*Distant metastasis															
Negative	162	4	96	60	2	88.88	−6.805	0.000	13	89	52	8	92.71	−4.694	0.000
Positive	40	2	2	20	16	152.60			1	9	20	10	137.10		

### Inactivation of Lyn inhibited CD24-induced ERK1/2 activation

**Figure 4:**
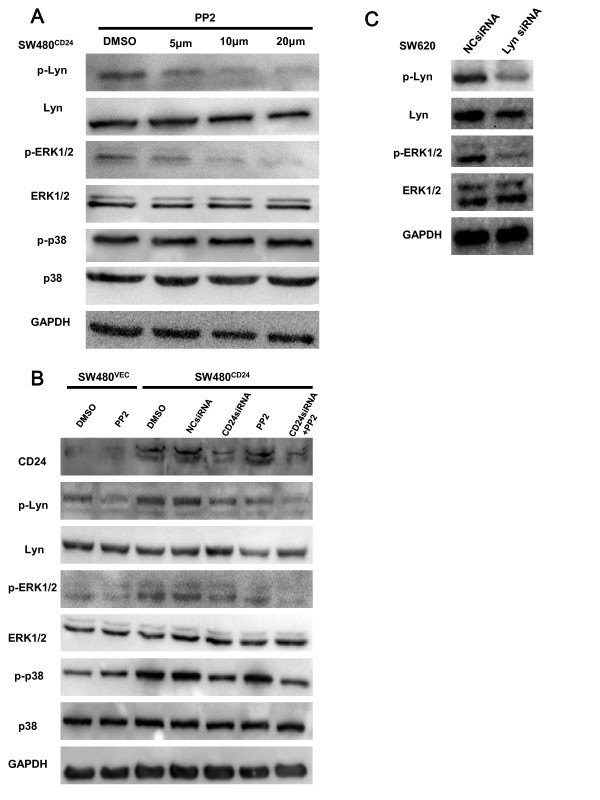
**CD24-induced ERK1/2 activation was regulated by the activation of Lyn. A.** SW480^CD24^ cells were treated with increasing doses of PP2 (0 μM, 5 μM, 10 μM, and 20 μM) for 30 min and the medium was renewed. Cell lysates were prepared 24 h later after treatment, and the level of phospho-Lyn, phospho-ERK and phospho-p38 MAPK were measured by Western blot analysis. The decreased expression peaked at 20 μM PP2 (lane 4) and the dose of 10 μM PP2 was selected for further studies. **B.** SW480^CD24^ cells were transfected with vehicle control (NCsiRNA) or CD24 specific siRNA 24 h prior to PP2 (10 μM) treatment. The cells were treated with PP2 for 30 min and harvested 24 h after treatment. The expression of CD24, as well as the level of phospho-Lyn, phospho-ERK1/2 and phospho-p38 MAPK, was assessed by Western blot analysis. **C.** SW620 cells were transfected with vehicle control (NCsiRNA) or Lyn specific siRNA and lysed 24 h after treatment. ERK1/2 phosphorylation was assessed by Western blot analysis.

In our previous study, we found that CD24 induced the activation of ERK1/2 and p38 MAPK. To explore the mechanism underlying CD24-induced ERK1/2 and p38 MAPK activation, PP2, a specific inhibitor of SFKs, was used in this study. We exposed SW480^CD24^ cells to a dose range of PP2, as indicated in Figure 4A, followed by Western blot analysis of the phosphorylation of Lyn, Erk1/2, and p38 MAPK.

Our data showed that the phosphorylation level of Lyn and ERK1/2 decreased in PP2 treated SW480^CD24^ cells in a dose-dependent manner and peaked at 20 μM of PP2 (Figure 4A). However, the phosphorylation level of p38 MAPK was not impacted by PP2. The IC50 occurred at 10 μM PP2 in the preliminary study (data not shown); therefore, we chose 10 μM as a standard dose in this study.

CD24 was depleted by a specific siRNA for CD24, which led to the reduction of the phosphorylation of Lyn, ERK1/2 and p38 MAPK in SW480^CD24^ cells (Figure 4B). CD24 specific siRNA and PP2 synergistically inactivated ERK1/2, but not p38MAPK in both SW480^CD24^ (Figure 4B) and SW620 cells (data not shown). We used another CD24 siRNA (CD24 siRNA-2) to confirm these data. CD24 siRNA-2 showed a similar effect (Additional file 1: Figure S2). To identify the role of Lyn in the CD24-mediated activation of ERK, Lyn was also depleted with a Lyn specific siRNA. The phosphorylation level of ERK1/2 was dramatically decreased in Lyn depleted SW620 cells (Figure 4C), suggesting that inactivation of Lyn could attenuate CD24-induced ERK1/2 activation. Means ± standard errors (SE) for three independent experiments for Figure 4 are shown in Additional file 1: Figure S3.

### Expression of ectopic CD24 enhanced the interaction of Lyn and ERK1/2 and induced the nuclear translocation of Lyn

**Figure 5:**
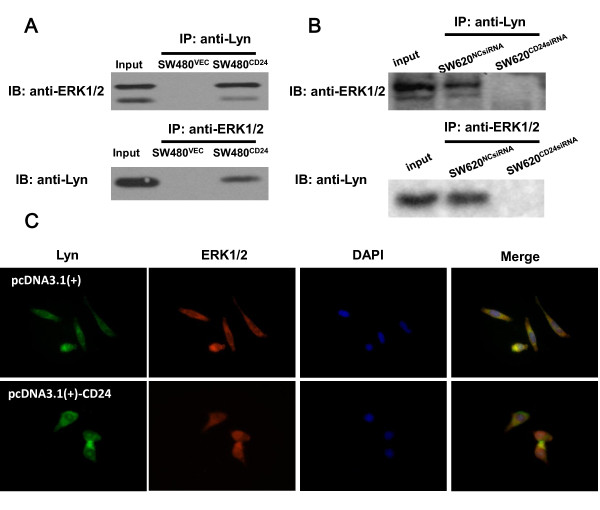
**The interaction between Lyn and ERK1/2. A.** The interaction between Lyn and ERK1/2 was detected in SW480^CD24^ and SW480^VEC^ cells. Cell lysates were immunoprecipitated by an anti-Lyn antibody and assayed by Western blotting with an anti-ERK1/2 antibody. Normal rabbit IgG served as a control. **B.** SW620 cells were transiently transfected with CD24 siRNA. Cell lysates were immunoprecipitated by an anti-Lyn antibody and assayed by Western blotting analysis with a ERK1/2 antibody. Pictures are representatives of two or three independent experiments with identical results. **C.** Immunofluorescent staining of SW620 cells transfected with CD24 siRNA. SW620 cells were transiently transfected with CD24siRNA or vehicle control for 24 h. Cytosolic and nucleus location of Lyn (green) and ERK1/2 (red) were examined by primary antibodies followed by FITC-conjugated secondary antibodies and visualized under fluorescent microscopy (original magnification = 630×, Oil). The nucleus was stained with 4, 6-diamidino-2-phenylindole (DAPI; blue). phospo-Lyn and phospho-ERK1/2 signals were greater and intense in the nucleus.

SFKs are the upstream modulator of MAP kinases in several receptor signaling pathways. To examine the interaction of Lyn and ERK1/2, as well as the involved regulation of CD24, CO-IP was performed for the interaction of Lyn and ERK. As shown in Figure 5A, the interaction between Lyn and ERK1/2 was identified in SW480^CD24^, but not in SW480^VEC^ cells, indicating that CD24 could enhance the binding of Lyn to ERK1/2. Our data also showed that the interaction between Lyn and ERK1/2 in SW620 cells, a CD24 abundant cell line, was disrupted when CD24 was depleted by CD24 siRNA (Figure 5B). These data supported the hypothesis that Lyn interacted with ERK1/2 directly or indirectly in a CD24-dependent manner. Furthermore, we examined the effect of ectopically expressed CD24 on the location of Lyn-ERK1/2. Nuclear accumulations of Lyn and ERK1/2 were observed with an immunofluorescence microscopy in ectopic CD24 expressed cells (Figure 5C).

### The expression of CD24 and Lyn was associated with the poor prognosis of CRC patients, and CD24 was an independent prognostic factor of CRC

To investigate the prognostic value of CD24 and Lyn, the association of CD24 and Lyn with an overall survival was evaluated using Kaplan-Meier survival curves with the log-rank test. Seventy-four CRC patients were enrolled for this analysis. The follow-up time ranged from 1 to 60 months. The median survival time of the group with low expression of Lyn was 54.101 months, and the cumulative 1-, 3- and 5-year survival rates were 90 %, 85 % and 83 %, respectively. The median survival time of the high expression group was 36.641 months, and the 1-, 3- and 5-year survival rates were 61 %, 54 % and 50 %, respectively. The difference between the groups was significant. The median survival time of the group with low expression of CD24 was 55.299 months, and the 1-, 3- and 5-year survival rates were 95 %, 91 % and 86 %, respectively, while the survival rate of high expression group was 36.324 months.

**Figure 6:**
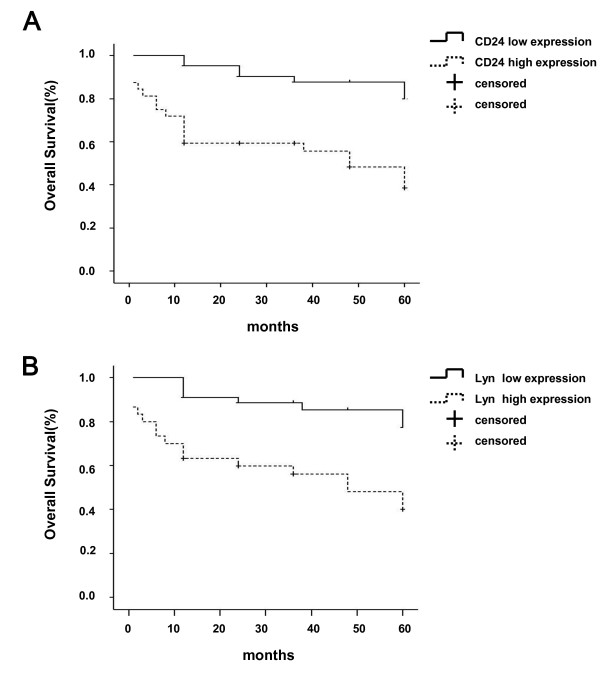
**Kaplan-Meier survival curves of 74 CRC patients relating to the status of CD24 (A) and Lyn (B) expression.** The log-rank test was used to calculate P values (CD24: P = 0.020).

The univariate survival analysis indicated that the survival rates of patients with low expression of CD24 ( $\chi_{}^{2}$=14.546, P = 0.000) or Lyn ( $\chi_{}^{2}$=12.559, P = 0.000) was higher than that of patients with high expression (log rank, P < 0.05; Figure 6 and Table 3). Other factors that were associated with tumor prognosis included tumor size, tumor stage, and lymph node and distant metastasis (P < 0.05).

**Table 3:** Univariate survival analysis of 74 CRC patients

**Factors**	**case(n)**	$\chi_{}^{2}$	***P***
Sex			
Males	45/15	0.027	0.869
Female	29/10		
Age			
≤60y	37/13	0.103	0.748
>60y	37/12		
Localization			
Right colon	14/3	4.530	0.104
Left colon	18/8		
Rectum	42/14		
Tumor size			
≤5cm	50/21	4.777	0.029
>5cm	24/4		
Grade			
G1	36/10	4.867	0.088
G2	26/8		
G3	12/7		
Invasion Depth			
T1	8/1	2.297	0.513
T2	35/11		
T3	28/12		
T4	3/1		
Tumor stage			
I	8/0	36.033	0.000
II	26/1		
III	21/10		
IV	19/14		
Lymph node			
Negative	42/8	13.972	0.000
Positive	32/17		
Distant metastasis			
Negative	55/10	50.535	0.000
Positive	19/15		
CD24			
low	42/7	14.456	0.000
high	32/18		
Lyn			
low	44/8	12.559	0.000
high	30/17		

The Cox multivariate analysis showed that the expression of CD24 (P = 0.020, RR = 2.918, 95 % CI: 1.188-7.166), but not Lyn expression, could be used as an independent prognostic factor. Our data also showed that tumor distant metastasis (P = 0.008, RR = 4.396, 95 % CI: 1.475-13.105) and tumor stage (P = 0.019, RR = 2.289, 95 % CI: 1.146 - 4.573) were also independent prognostic factors for CRC patients (Table 4.).

**Table 4:** Multivariate analysis of prognostic factors in 74 CRC patients

	**B**	**SE**	**Wald**	**df**	***P***	**RR**	**95.0 % CI for Exp(B)**	
							**Lower**	**Upper**
Tumor stage	0.828	0.353	5.499	1	0.019	2.289	1.146	4.573
Distant Metastasis	1.481	0.557	7.059	1	0.008	4.396	1.475	13.105
CD24	1.071	0.458	5.456	1	0.020	2.918	1.188	7.166

## Discussion

It is reported that SFKs are over-expressed and activated in a large number of human malignancies. SFKs have been found to be associated with the development of cancer and progression to distant metastasis, including CRC [18,21]. More interesting, GPI-anchored proteins-related signaling transduction is also mediated by SFKs [22]. In our previous study, we found that ERK1/2 was involved in CD24-induced CRC cell proliferation [7] and invasion. In the present study, we aimed to investigate the role of SFKs in this cellular process. Therefore, we first detected the effects of CD24 on the activity of SFKs in CRC cells by Western blotting. We found that CD24 could increase the expression of phospho-Lyn (Y396), but not phospho-Src, phospho-Fyn or phospho-lck in CRC cells, which indicated a tumor-type dependent induction. Three research groups [12,23,24] reported that CD24 interacted with Lyn directly or indirectly in monocytic ESb-MP cells, in the erythroleukaemia cell line K562 and in the Burkitt’s lymphoma (BL)-derived cell line P32/SH. Therefore, we hypothesized that there was an interaction between CD24 and Lyn directly or indirectly in CRC. Through CO-IP assays, we identified this interaction in CRC, and this might be the first report of an interaction between CD24 and Lyn in solid tumors. To identify the interaction between CD24 and Lyn *in vivo*, we examined the expression of CD24 and Lyn by immunohistochemical staining on serial sections of CRC tissues. The results showed that the expression of CD24 was positively correlated with phospho-Lyn expression in 202 CRC patients.

Mierke *et al.* found that CD24 enhanced human lung cancer cell invasion through increased generation or transmission of contractile forces [25]. In the present study, we also found that overexpression of CD24 could increase the cell invasion ability. Furthermore, we investigated the role of Lyn in CD24- induced CRC cell invasion. After treating cells with PP2, a specific inhibitor of SFKs, CD24-induced CRC cell invasion was abrogated, suggesting that Lyn was involved in CD24-induced cell invasion. SFKs are known to be ubiquitously distributed in the cell membrane, but Src is located in perinuclear membranes, endosomes and possibly even the nucleus, implicating the involvement of Src in nuclear-signal transduction events [26]. The regulation of nucleocytoplasmic distribution of Lyn contributes to the association of Lyn with other upstream or downstream signaling molecules. To elucidate the underlying mechanisms in this process, we examined the subcellular localization of Lyn by immunoflourescence staining. In SW480^VEC^ cells, positive cellular signals of Lyn was predominant in the cytoplasm, and over-expression of CD24 resulted in a cytosolic decrease and a nuclear accumulation of Lyn. The nuclear translocation strongly implied that the regulation of gene transcription of Lyn might be involved, which should be clarified in further studies. In addition, we found that Lyn and CD24 were expressed in different tumor stages of CRC and the expression level of CD24 and Lyn was positively correlated with tumor grade, tumor stage, invasion depth, and lymph node and distant metastasis.

SFKs are the upstream modulator of MAP kinases in several receptor signaling pathways. Therefore, it is possible that CD24 directly or indirectly interacts with Lyn and affects the activity of ERK1/2. We found that the depletion of CD24 and Lyn, by specific siRNA or inactivation of Lyn by its specific inhibitor PP2, could reduce the phosphorylation level of ERK1/2 respectively or synergistically. CO-IP assays showed that Lyn interacted with ERK1/2 in a CD24 high expression cell line and the Lyn-ERK interaction was disrupted by the depletion of CD24, suggesting that Lyn was involved in CD24-induced ERK1/2 activation in CRC cells. Therefore, it is possible that CD24 directly or indirectly induces the activation of Lyn and promotes the interaction and nuclear translocation of Lyn, leading to cell invasion. To better understand the Lyn-ERK1/2 interaction and the related regulation, further investigations are required to characterize the interaction site or structure among CD24, Lyn and ERK1/2 on the membrane, such as glycolipid-enriched membrane (GEM) domains or nucleus.

The studies on the roles of CD24 and Lyn in CRC invasion provided potential targets for CRC diagnosis and prognosis. However, the clinicopathologic significance of CD24 is still controversial [7,27,28] and very few studies have elucidated the relationship between Lyn and clinicopathological characteristics of CRC. Sagiv *et al.*[6] showed that CD24 was expressed in 90.7 % of adenomas and 86.3 % of CRC. In addition, recent studies suggested that CD24 was a promising therapeutic target in cancers of the gastrointestinal tract and bladder cancer metastasis [29-31]. In our study, strong expression of cytoplasmic CD24 correlated significantly to shortened survival of CRC patients without distant metastases [28]. However, studies from Ahmed *et al.*[32] and Choi *et al.*[33] showed early CD24 up-regulation and nuclear expression, but it was not a prognostic marker for CRC. Hao *et al.*[21] showed that Lyn was significantly correlated with overall survival in CRC patients. In the present study, the Cox multivariate analysis showed that CD24, tumor distant metastasis and tumor stage were independent prognostic factors of CRC patients. In contrast, Lyn was not an independent prognostic factor of CRC, which is different from previously reported studies.

In this study, we showed that Lyn was involved in CD24-induced ERK1/2 activation and CRC cell invasion *in vitro*. *In vivo*, we found aberrant CD24 and Lyn expression in the majority of the CRC tissues and a significant correlation between CD24 and Lyn. CD24 was identified as an independent prognostic factor of CRC, and the expression of CD24 was associated with the activation of Lyn and ERK1/2, which might be a novel mechanism related to CD24-mediated regulation of CRC development.

## Materials and Methods

### Reagents and antibodies

RPMI-1640 medium and FBS were purchased from Life Technologies (Grand Island, NY). G418 was obtained from Calbiochem (San Diego, CA). The PP2 inhibitor was purchased from Sigma (St. Louis, MO). ERK1/2 (137 F5), phospho-ERK1/2 (Thr202/Tyr204), p38 MAPK, phospho-p38 MAPK (Thr180/Tyr186), SAPK/JNK, phospho-SAPK/JNK (Thr183/Tyr185), Lyn (C13F9), Src, and phospho-Src (TYR416) antibodies were purchased from Cell Signaling Technology (Danvers, MA). The phospho-Lyn (Y396) antibody was purchased from Abcam (Cambridge, MA). CD24 (C-20), GAPDH, phospho-Fyn (Thr12), Fyn, phospho-lck (Tyr394) and lck antibodies, FITC-antibody, and Lyn siRNA were purchased from Santa Cruz Biotechnology (Santa Cruz, CA).

### Tissue samples

Formalin-fixed, paraffin-embedded tissue samples from 202 (110 men and 92 women) primary CRC patients were randomly obtained and processed by routine clinical histopathological methods. The patients had a mean age of 57 years (range 18-87 years) and a median age of 59 years. The pathological tumor stages were determined according to the TNM classification of the American Joint Committee on Cancer Criteria, and the World Health Organization (WHO) classification of tumors was used to determine the histological grade. Clinical follow-up data were available for 74 patients, while 128 patients were excluded for lack of information. The study was carried out in accordance with the institutional ethical guidelines and was approved by the Medical Ethics Committee of Southern Medical University. Informed consent was obtained from all patients.

### Cell culture and treatments

SW480 and SW620 cell lines were obtained from American Type Culture Collection (Rockville, MD). SW480^CD24^ and SW480^VEC^ cells were established, as described in a previous study [7]. Cells were maintained in RPMI-1640 medium supplemented with 10 % FBS (complete medium) and 1000 μg/ml G418 at 37°C with an atmosphere of 5 % CO_2_ and subcultured by digestion with 0.05 % trypsin. For PP2 treatment, cells were seeded in serum-free medium overnight, followed by the addition of PP2 as indicated for 30 min at 37°C. Medium containing the PP2 inhibitor was renewed after 30 min of incubation.

### RT-PCR

Total RNA was isolated from cells using Trizol (Invitrogen). Next, 2 μg of RNA sample was subjected to reverse transcription using a RevertAid First Strand cDNA Synthesis Kit (K1622, Fermentas, MBI, Vilnius, Lithuania). The primer pairs for CD24 were 5'-TAGGTACCACTATGGGCAGAGCAATGG-3' (forward) and 5'-CCGGAATTCCGTTAAGAGTAGAGATGC-3' (reverse). The primer pairs for GAPDH were 5’-GTCAACGGATTTGGTCGTATTG-3’ (forward) and 5’- CTCCTGGAAGATGGTGATGGG-3’ (reverse). PCR was initiated by 5 min incubation at 94°C, 36 cycles of denaturation at 94°C for 45 s, annealing at 56°C and extension at 72°C for 50 s, and ended after a 7 min extension at 72°C using a PCR kit (SBS, Beijing, China). The experiments were repeated twice and GAPDH mRNA was amplified simultaneously as an internal control.

### Preparation of siRNA and transfection

A siRNA duplex targeting CD24 and a negative control sequence were designed and synthesized by GenePharma Co. (Shanghai, China). The target sequence of the CD24 siRNA duplex was as follows: CD24 siRNA sense 5'-GAUUUAUUCCA GUGAAACATT-3' and antisense 5'-UGUUUCACUGGAAUA AAUCTG-3'. The negative control sequence was as follows: sense 5'-CUACCUAUGCAGAUUU AUUdTdT-3' and antisense 5'-AAUAAAUCUGCAUAGGUAGdTdT-3'. BLAST research confirmed the unique sequence of the siRNA. Lyn siRNA was purchased from Santa Cruz Biotechnology (sc-29393, Santa Cruz, CA). The specific and negative control siRNAs were transfected into SW480^CD24^ and/or SW620 cells 24 h prior to Western blotting analyses or cell invasion assays using lipofectamine^TM^ 2000 reagent according to the manufacturer’s instructions.

### Western blotting analysis

Whole cell lysates were prepared as described previously [34]. Equal aliquots of total cell lysates (30 μg) were electrophoresed on denaturing sodium dodecy1 sulfate-polyacrylamide gel electrophoresis (SDS-PAGE) gels (5 % stacking gel and 8 %-12 % resolving gel). The proteins were then transferred to polyvinylidene difluoride (PVDF) membranes (Millipore, Bedford, MA). The blots were probed with primary antibodies followed by the horseradish peroxidase-conjugated secondary antibody. Antigen-antibody complexes were visualized using an enhanced chemiluminescence system (Amersham Biosciences, Little Chalfont Buchkinghamshire, UK). GAPDH served as the loading control.

### Immunofluorescence

Immunofluorescent staining was performed as described [35]. In brief, cells grown on cover glass were fixed in phosphate-buffered saline (PBS) containing 4 % paraformaldehyde for 30 min at room temperature. Nonspecific binding was blocked by incubation with 1 % bovine serum albumin (BSA). Cells were subsequently reacted with an appropriate primary antibody for 1 h, then washed and stained with TR-or fluorescein isothiocyanate-conjugated second antibodies. After mounting with Prolong Antifade™ reagent (Molecular Probes), the cells were visualized under an Olympus CKX41 fluorescence microscope (Olympus, Tokyo, Japan).

### Co-IP

As described previously [36], 2 mg of cell lysates from control or transfected cells were incubated with 3 μg of primary antibody overnight at 4°C on an oscillation shaker. Then, 50 μl of suspension, with a 1:1 ratio of protein A-Sepharose beads, were added and incubated for 2 h at 4°C with gentle rotation. After extensive washing, precipitates were subjected to Western blotting analyses for detection of potential interacting proteins. Normal rabbit IgG served as a negative control.

### Cell invasion assay

Cell invasion was assessed using an invasion assay kit (ECM551, Millipore, Bedford, MA) [37] according to manufacturer’s instructions. The cells, which were treated according to different experimental purposes, were suspended in serum free RPMI-1640 medium at a density of 1.0 × 10^6^/ml. Next, 300 μl of the prepared cell suspension and 500 μl of RPMI-1640 containing 10%FBS were respectively added in each insert and the matched lower chamber and incubated for additional 24 h. Non-invaded cells were removed with cotton swabs. Invasive cells were stained with 0.2 % crystal violet and counted under a microscope. Six random fields for each insert were counted (per HPF × 200).

### Immunohistochemical analysis and evaluation

The Medical Ethics Committee of Southern Medical University approved our experimental protocols. Paraffin-embedded tissue blocks were cut into 4 μm sections and transferred to glass slides. The slides were deparaffinized with xylene, rehydrated with ethanol, washed, and subjected to microwave retrieval in a citrate buffer. Sections were then immersed in 3 % hydrogen peroxide to block endogenous peroxidase activity and incubated with primary antibodies at 4°C overnight followed by incubation with goat or rabbit serum for 30 min the next day. Subsequent steps utilized the UltraSensitive^TM^ SP kit (KIT-0310, Maxin_bio, Fuzhou, China) according to the manufacturer’s instructions. The expressions of CD24 and Lyn were visualized using DAB and counterstained with hematoxylin. Tissues were considered “positive” when 10 % or more of the cancer cells were positively stained [38]. For quantitative analysis, the ratio of positively stained cells to all tumor cells in five random areas at 200X and/or 400X magnification was recorded.

### Statistical analysis

Statistical analysis was performed using the software package SPSS 13.0. ANOVA provided a statistical test used to determine the results of cell invasion assay. Non-parametric Kruskal-Wallis or Mann–Whitney tests were used to test the differences between subgroups of clinic-pathological parameters, whereas the correlations of the expression of different proteins were evaluated by the Spearman rank-order correlation coefficient. Kaplan-Meier and Log-rank tests analyzed the univariate prognostic analysis, qualifying into the COX proportional hazards model for multivariate analysis. The level of significance was defined as *P* < 0.05.

## Abbreviations

BSA, bovine serum albumin; Co-IP, co-immunoprecipitation; CRC, colorectal cancer; ERK1/2, extracellular signal-regulated kinases 1 and 2; GEM, glycolipid-enriched membrane; PBS, phosphate-buffered saline; PVDF, polyvinylidene difluoride; SDS-PAGE, sodium dodecy1 sulfate-polyacrylamide gel electrophoresis; SFKs, Src family kinases; WHO, World Health Organization.

## Competing interests

The authors declare that they have no competing interests.

## Author's Contributions

NS and LP participated in the design of the study, and carried out the experiment, and performed the statistical analysis. BX, YZ, AX and JW participated in the experiment and drafted the manuscript. XW and BJ conceived of the study, and participated in its design and coordination and edited the manuscript. All authors read and approved the final manuscript.

## Supplementary Material

Additional file 1 **Supplementary figures.** Figure S1: Densitometry results of Figure 1A; Figure S2: CD24 siRNA-2 results; Figure S3: Means ± standard errors (SE) for three independent experiments for Figure 4. Click here for file

## References

[B1] Kristiansen G Sammar M Altevogt P Tumour biological aspects of CD24, a mucin-like adhesion molecule J Mol Histol 2004 35 255 262 15339045 10.1023/b:hijo.0000032357.16261.c5

[B2] Kristiansen G Winzer KJ Mayordomo E Bellach J Schluns K Denkert C Dahl E Pilarsky C Altevogt P Guski H Dietel M CD24 expression is a new prognostic marker in breast cancer Clin Cancer Res 2003 9 4906 4913 14581365

[B3] Kristiansen G Pilarsky C Pervan J Sturzebecher B Stephan C Jung K Loening S Rosenthal A Dietel M CD24 expression is a significant predictor of PSA relapse and poor prognosis in low grade or organ confined prostate cancer Prostate 2004 58 183 192 10.1002/pros.10324 14716744

[B4] Agrawal S Kuvshinoff BW Khoury T Yu J Javle MM LeVea C Groth J Coignet LJ Gibbs JF CD24 expression is an independent prognostic marker in cholangiocarcinoma J Gastrointest Surg 2007 11 445 451 10.1007/s11605-007-0091-5 17436128 PMC1852393

[B5] Chou YY Jeng YM Lee TT Hu FC Kao HL Lin WC Lai PL Hu RH Yuan RH Cytoplasmic CD24 expression is a novel prognostic factor in diffuse-type gastric adenocarcinoma Ann Surg Oncol 2007 14 2748 2758 10.1245/s10434-007-9501-x 17680316

[B6] Sagiv E Memeo L Karin A Kazanov D Jacob-Hirsch J Mansukhani M Rechavi G Hibshoosh H Arber N CD24 is a new oncogene, early at the multistep process of colorectal cancer carcinogenesis Gastroenterology 2006 131 630 639 10.1053/j.gastro.2006.04.028 16890615

[B7] Wang W Wang X Peng L Deng Q Liang Y Qing H Jiang B CD24-dependent MAPK pathway activation is required for colorectal cancer cell proliferation Cancer Sci 2010 101 112 119 10.1111/j.1349-7006.2009.01370.x 19860845 PMC11159715

[B8] Smith SC Oxford G Wu Z Nitz MD Conaway M Frierson HF Hampton G Theodorescu D The metastasis-associated gene CD24 is regulated by Ral GTPase and is a mediator of cell proliferation and survival in human cancer Cancer Res 2006 66 1917 1922 10.1158/0008-5472.CAN-05-3855 16488989

[B9] Bretz N Noske A Keller S Erbe-Hofmann N Schlange T Salnikov AV Moldenhauer G Kristiansen G Altevogt P CD24 promotes tumor cell invasion by suppressing tissue factor pathway inhibitor-2 (TFPI-2) in a c-Src-dependent fashion Clin Exp Metastasis 2011 10.1007/s10585-011-9426-4 21984372

[B10] Friederichs J Zeller Y Hafezi-Moghadam A Grone HJ Ley K Altevogt P The CD24/P-selectin binding pathway initiates lung arrest of human A125 adenocarcinoma cells Cancer Res 2000 60 6714 6722 11118057

[B11] Chen GY Tang J Zheng P Liu Y CD24 and Siglec-10 selectively repress tissue damage-induced immune responses Science 2009 323 1722 1725 10.1126/science.1168988 19264983 PMC2765686

[B12] Zarn JA Zimmermann SM Pass MK Waibel R Stahel RA Association of CD24 with the kinase c-fgr in a small cell lung cancer cell line and with the kinase lyn in an erythroleukemia cell line Biochem Biophys Res Commun 1996 225 384 391 10.1006/bbrc.1996.1184 8753773

[B13] Baumann P Thiele W Cremers N Muppala S Krachulec J Diefenbacher M Kassel O Mudduluru G Allgayer H Frame M Sleeman JP CD24 interacts with and promotes the activity of c-src within lipid rafts in breast cancer cells, thereby increasing integrin-dependent adhesion Cell Mol Life Sci 2011 10.1007/s00018-011-0756-9 PMC11114536 21710320

[B14] Pazdrak K Schreiber D Forsythe P Justement L Alam R The intracellular signal transduction mechanism of interleukin 5 in eosinophils: the involvement of lyn tyrosine kinase and the Ras-Raf-1-MEK-microtubule-associated protein kinase pathway J Exp Med 1995 181 1827 1834 10.1084/jem.181.5.1827 7722458 PMC2192005

[B15] Bjorge JD Jakymiw A Fujita DJ Selected glimpses into the activation and function of Src kinase Oncogene 2000 19 5620 5635 10.1038/sj.onc.1203923 11114743

[B16] Yeatman TJ A renaissance for SRC Nat Rev Cancer 2004 4 470 480 10.1038/nrc1366 15170449

[B17] Janas ML Hodgkin P Hibbs M Tarlinton D Genetic evidence for Lyn as a negative regulator of IL-4 signaling J Immunol 1999 163 4192 4198 10510355

[B18] Choi YL Bocanegra M Kwon MJ Shin YK Nam SJ Yang JH Kao J Godwin AK Pollack JR LYN is a mediator of epithelial-mesenchymal transition and a target of dasatinib in breast cancer Cancer Res 2010 70 2296 2306 10.1158/0008-5472.CAN-09-3141 20215510 PMC2869247

[B19] Park SI Zhang J Phillips KA Araujo JC Najjar AM Volgin AY Gelovani JG Kim SJ Wang Z Gallick GE Targeting SRC family kinases inhibits growth and lymph node metastases of prostate cancer in an orthotopic nude mouse model Cancer Res 2008 68 3323 3333 10.1158/0008-5472.CAN-07-2997 18451159

[B20] Montero JC Seoane S Ocana A Pandiella A Inhibition of SRC family kinases and receptor tyrosine kinases by dasatinib: possible combinations in solid tumors Clin Cancer Res 2011 17 5546 5552 10.1158/1078-0432.CCR-10-2616 21670084

[B21] Hao JM Chen JZ Sui HM Si-Ma XQ Li GQ Liu C Li JL Ding YQ Li JM A five-gene signature as a potential predictor of metastasis and survival in colorectal cancer J Pathol 2010 220 475 489 20077526 10.1002/path.2668

[B22] Sammar M Aigner S Altevogt P Heat-stable antigen (mouse CD24) in the brain: dual but distinct interaction with P-selectin and L1 Biochim Biophys Acta 1997 1337 287 294 10.1016/S0167-4838(96)00177-X 9048906

[B23] Sammar M Gulbins E Hilbert K Lang F Altevogt P Mouse CD24 as a signaling molecule for integrin-mediated cell binding: functional and physical association with src-kinases Biochem Biophys Res Commun 1997 234 330 334 10.1006/bbrc.1997.6639 9177270

[B24] Suzuki T Kiyokawa N Taguchi T Sekino T Katagiri YU Fujimoto J CD24 induces apoptosis in human B cells via the glycolipid-enriched membrane domains/rafts-mediated signaling system J Immunol 2001 166 5567 5577 11313396 10.4049/jimmunol.166.9.5567

[B25] Mierke CT Bretz N Altevogt P Contractile Forces Contribute to Increased Glycosylphosphatidylinositol-anchored Receptor CD24-facilitated Cancer Cell Invasion J Biol Chem 2011 286 34858 34871 10.1074/jbc.M111.245183 21828044 PMC3186419

[B26] Hayakawa F Naoe T SFK-STAT pathway: an alternative and important way to malignancies Ann N Y Acad Sci 2006 1086 213 222 10.1196/annals.1377.002 17185518

[B27] Lee JH Kim SH Lee ES Kim YS CD24 overexpression in cancer development and progression: a meta-analysis Oncol Rep 2009 22 1149 1156 19787233 10.3892/or_00000548

[B28] Weichert W Denkert C Burkhardt M Gansukh T Bellach J Altevogt P Dietel M Kristiansen G Cytoplasmic CD24 expression in colorectal cancer independently correlates with shortened patient survival Clin Cancer Res 2005 11 6574 6581 10.1158/1078-0432.CCR-05-0606 16166435

[B29] Overdevest JB Thomas S Kristiansen G Hansel DE Smith SC Theodorescu D CD24 offers a therapeutic target for control of bladder cancer metastasis based on a requirement for lung colonization Cancer Res 2011 71 3802 3811 10.1158/0008-5472.CAN-11-0519 21482678 PMC4283788

[B30] Sagiv E Starr A Rozovski U Khosravi R Altevogt P Wang T Arber N Targeting CD24 for treatment of colorectal and pancreatic cancer by monoclonal antibodies or small interfering RNA Cancer Res 2008 68 2803 2812 10.1158/0008-5472.CAN-07-6463 18413748

[B31] Shapira S Shapira A Starr A Kazanov D Kraus S Benhar I Arber N An immunoconjugate of anti-CD24 and Pseudomonas exotoxin selectively kills human colorectal tumors in mice Gastroenterology 2011 140 935 946 10.1053/j.gastro.2010.12.004 21147107

[B32] Ahmed MA Al-Attar A Kim J Watson NF Scholefield JH Durrant LG Ilyas M CD24 shows early upregulation and nuclear expression but is not a prognostic marker in colorectal cancer J Clin Pathol 2009 62 1117 1122 10.1136/jcp.2009.069310 19946098

[B33] Choi D Lee HW Hur KY Kim JJ Park GS Jang SH Song YS Jang KS Paik SS Cancer stem cell markers CD133 and CD24 correlate with invasiveness and differentiation in colorectal adenocarcinoma World J Gastroenterol 2009 15 2258 2264 10.3748/wjg.15.2258 19437567 PMC2682242

[B34] Wang X Li M Wang J Yeung CM Zhang H Kung HF Jiang B Lin MC The BH3-only protein, PUMA, is involved in oxaliplatin-induced apoptosis in colon cancer cells Biochem Pharmacol 2006 71 1540 1550 10.1016/j.bcp.2006.02.011 16595125

[B35] Yamaguchi N Nakayama Y Urakami T Suzuki S Nakamura T Suda T Oku N Overexpression of the Csk homologous kinase (Chk tyrosine kinase) induces multinucleation: a possible role for chromosome-associated Chk in chromosome dynamics J Cell Sci 2001 114 1631 1641 11309195 10.1242/jcs.114.9.1631

[B36] Lee EJ Seo SR Um JW Park J Oh Y Chung KC NF-kappaB-inducing kinase phosphorylates and blocks the degradation of Down syndrome candidate region 1 J Biol Chem 2008 283 3392 3400 18056702 10.1074/jbc.M706707200

[B37] Albini A Iwamoto Y Kleinman HK Martin GR Aaronson SA Kozlowski JM McEwan RN A rapid in vitro assay for quantitating the invasive potential of tumor cells Cancer Res 1987 47 3239 3245 2438036

[B38] Jin SH Akiyama Y Fukamachi H Yanagihara K Akashi T Yuasa Y IQGAP2 inactivation through aberrant promoter methylation and promotion of invasion in gastric cancer cells Int J Cancer 2008 122 1040 1046 17957782 10.1002/ijc.23181

