# Correction: Lyn is involved in CD24-induced ERK1/2 activation in colorectal cancer

**DOI:** 10.1186/1476-4598-11-68

**Published:** 2012-09-14

**Authors:** Ning Su, Liang Peng, Bingqing Xia, Yingying Zhao, Angao Xu, Jing Wang, Xinying Wang, Bo Jiang

**Affiliations:** 1 Department of Gastroenterology, Nanfang Hospital, Southern Medical University, Guangzhou 510515, China; 2 Guangdong Provincial key laboratory of Gastroenterology, Guangzhou 510515, China; 3 Huizhou Medical Institute, Huizhou, Guangdong, 516001, China

## Correction

To further confirm that the inactivation of Lyn inhibited CD24-induced ERK1/2 activation, we submitted the results of another CD24siRNA (CD24siRNA-2) as the Additional file 1: Figure S2. After publication of the article [1] the authors noted that the wrong figure for Additional file 1: Figure S2 was submitted by mistake. The correct image was conducted in SW480^CD24^ using CD24siRNA-2 (new Additional file 1: attached). The authors apologise for any inconvenience caused.

## Supplementary Material

Additional file 1 **Figure S1: **Densitometry results of Figure 1A; **Figure S2: **CD24siRNA-2 results; **Figure S3:** Means ± standard errors (SE) for three independent experiments for Figure 4. Click here for file
