# Supplementary figures and images for: Lyn is involved in CD24-induced ERK1/2 activation in colorectal cancer
Source: Mol Cancer. 2012 Jun 26;11:43. doi: 10.1186/1476-4598-11-43 (PMC3464950; doi:10.1186/1476-4598-11-43)

**Fig.S1**

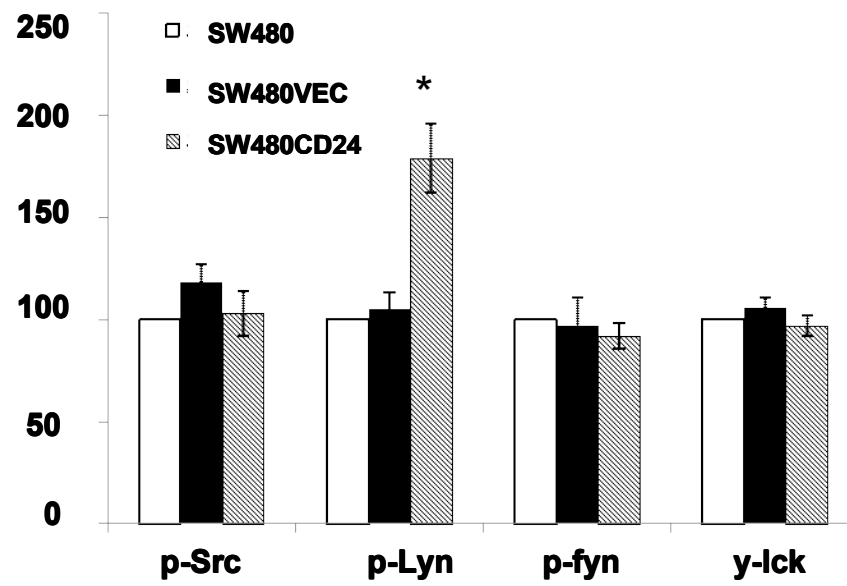

**Fig.S2**

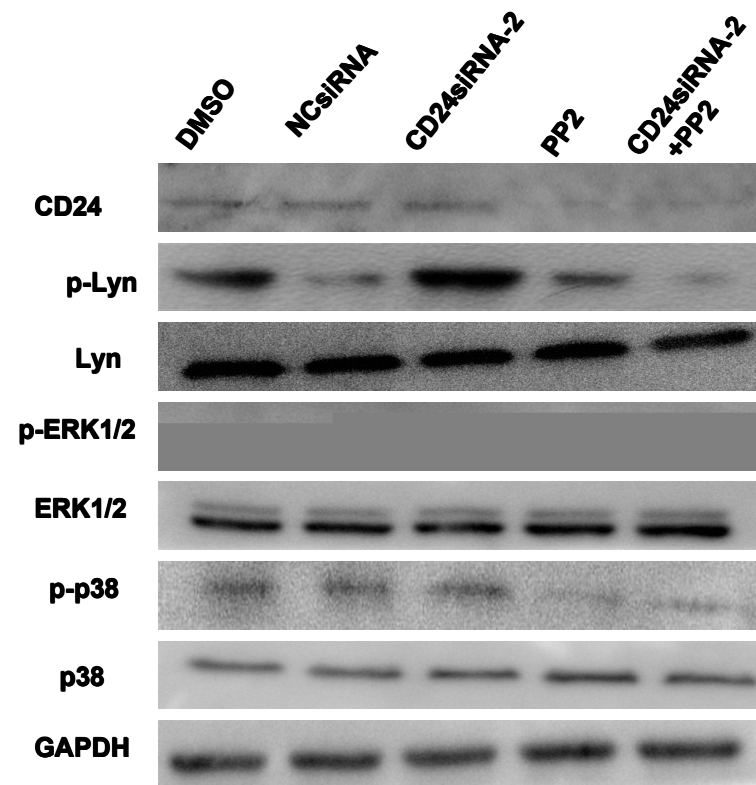

**Fig.S3**

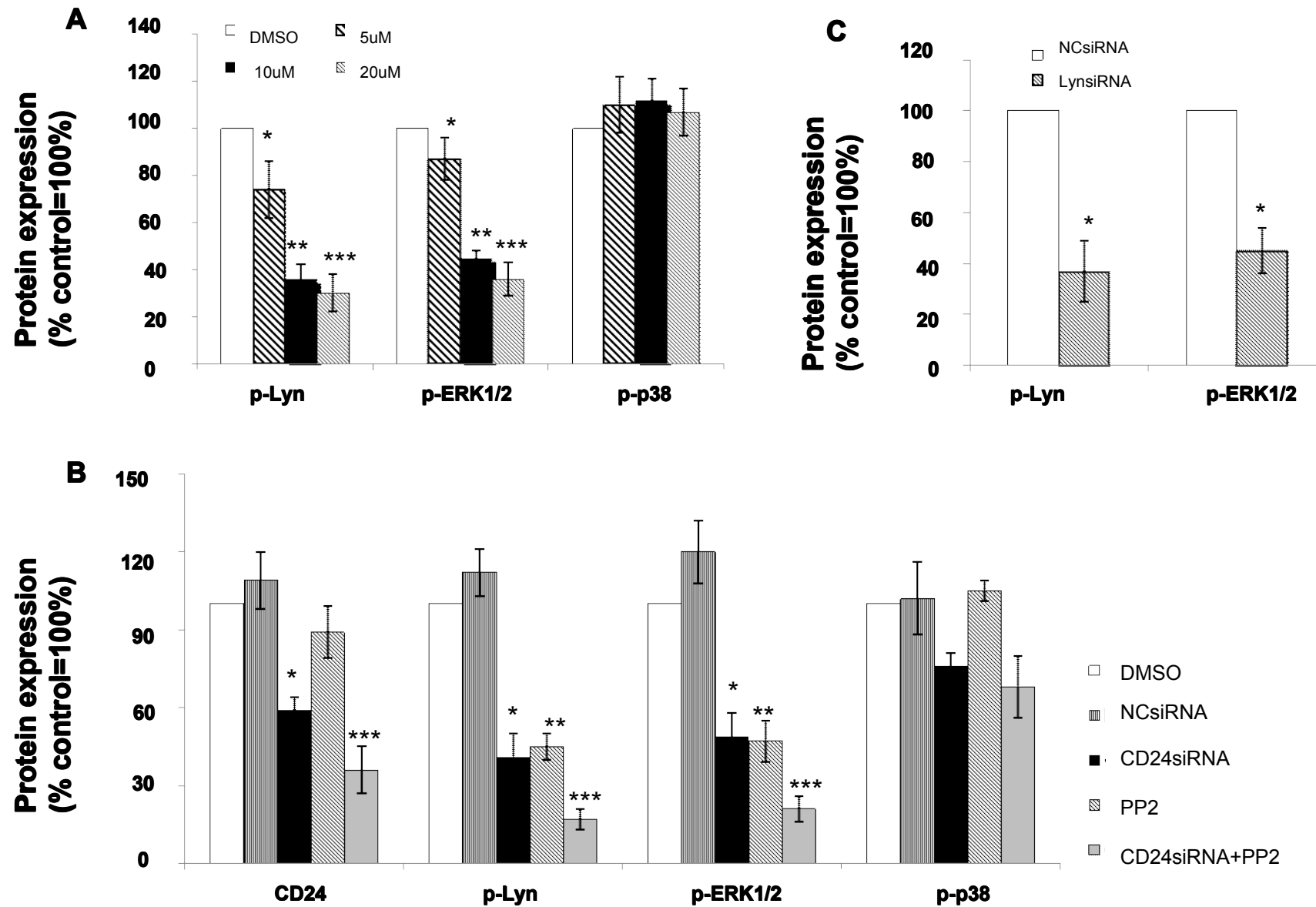

Supplement: Additional file 1 — Supplementary figures. Figure S1: Densitometry results of Figure 1A; Figure S2: CD24 siRNA-2 results; Figure S3: Means ± standard errors (SE) for three independent experiments for Figure 4. [file 1476-4598-11-43-S1.pdf]
