# Supplementary figures and images for: Correction: Lyn is involved in CD24-induced ERK1/2 activation in colorectal cancer
Source: Mol Cancer. 2012 Sep 14;11:68. doi: 10.1186/1476-4598-11-68 (PMC3527162; doi:10.1186/1476-4598-11-68)

**Fig.S1**

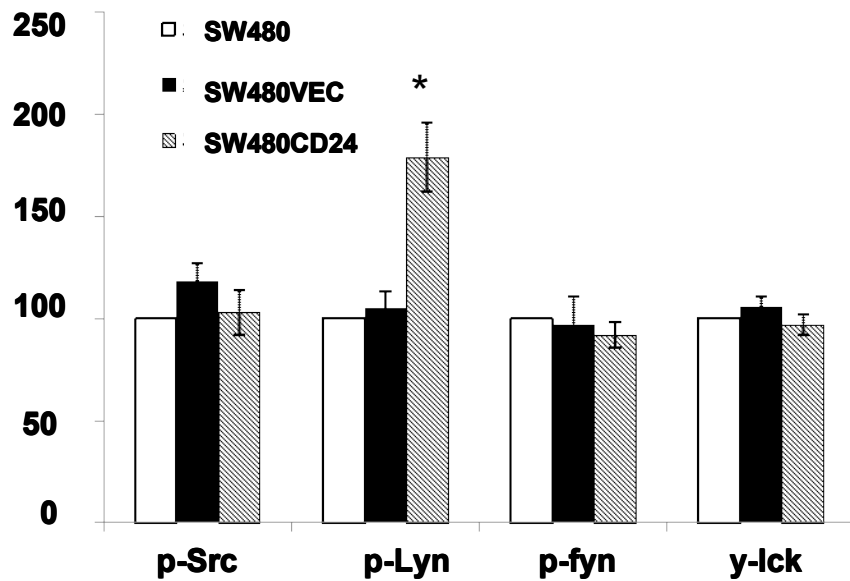

**Fig.S2**

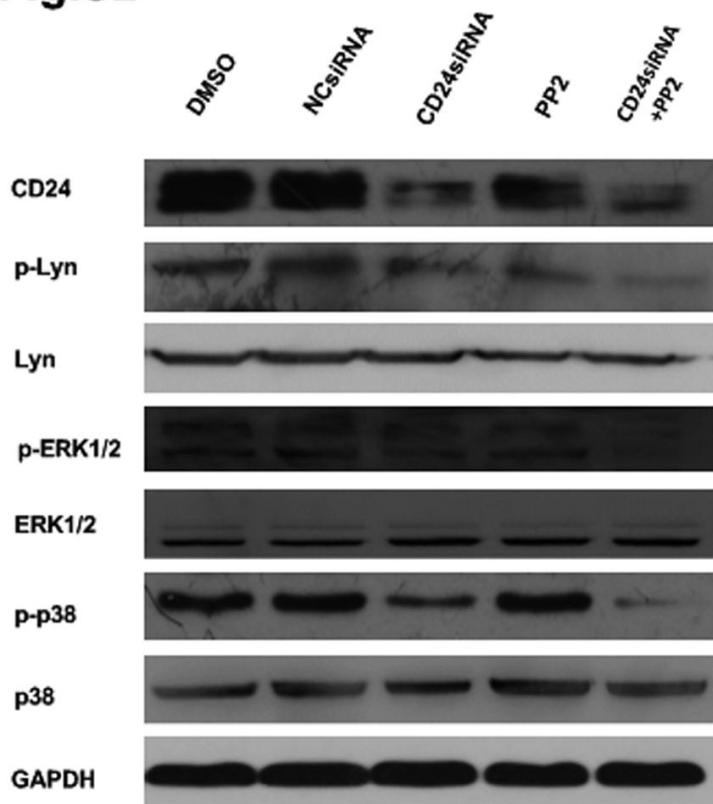

**Fig.S3**

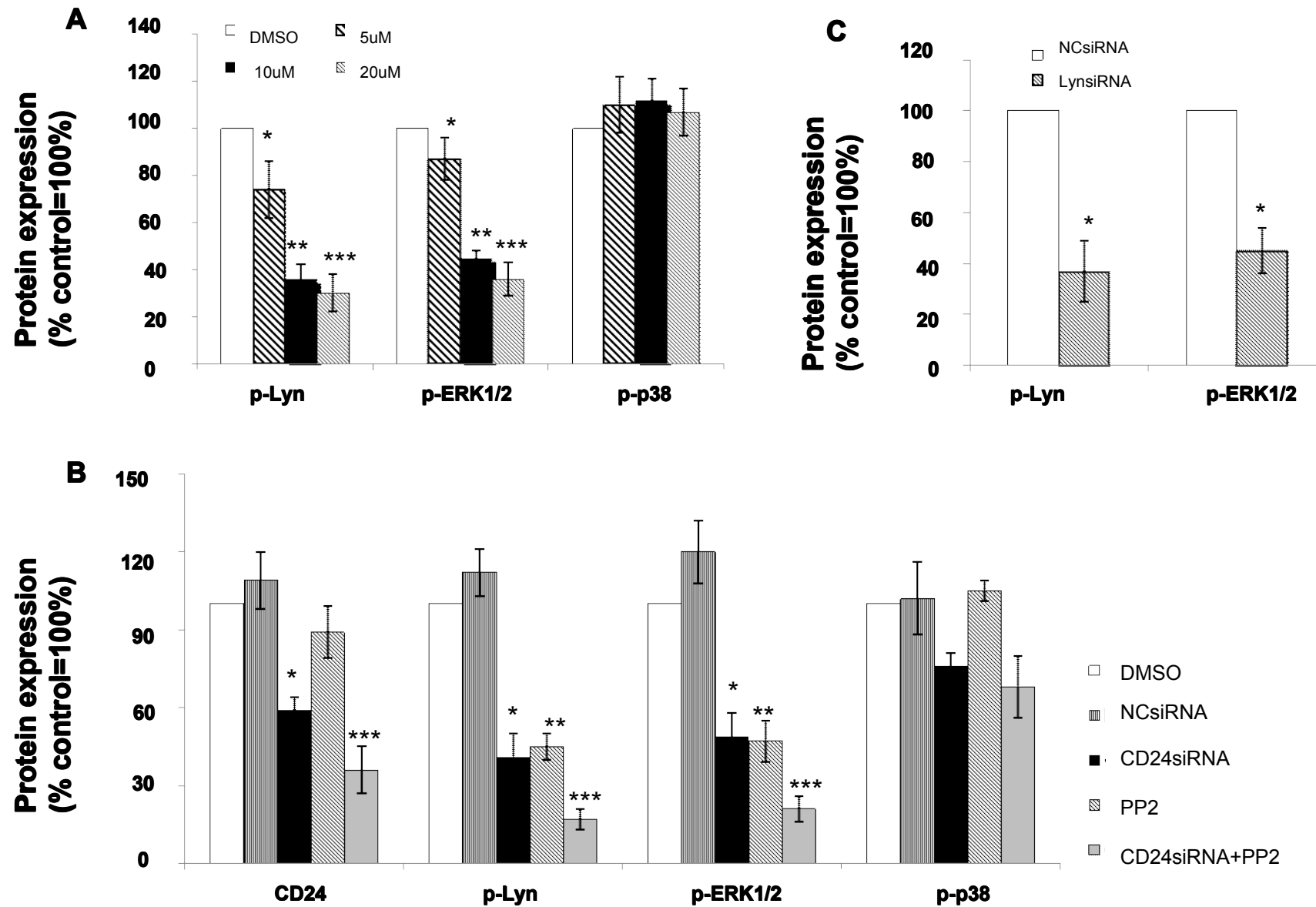

Supplement: Additional file 1 — Figure S1: Densitometry results of Figure 1A; Figure S2: CD24siRNA-2 results; Figure S3: Means ± standard errors (SE) for three independent experiments for Figure 4. [file 1476-4598-11-68-S1.pdf]
